# The Relationship between Body Mass Index and Mammographic Density during a Premenopausal Weight Loss Intervention Study

**DOI:** 10.3390/cancers13133245

**Published:** 2021-06-29

**Authors:** Emma C. Atakpa, Adam R. Brentnall, Susan Astley, Jack Cuzick, D. Gareth Evans, Ruth M. L. Warren, Anthony Howell, Michelle Harvie

**Affiliations:** 1 Centre for Cancer Prevention, Wolfson Institute of Preventive Medicine, Barts and The London School of Medicine and Dentistry, Queen Mary University of London, London EC1M 6BQ, UK; e.c.atakpa@qmul.ac.uk (E.C.A.); a.brentnall@qmul.ac.uk (A.R.B.); j.cuzick@qmul.ac.uk (J.C.); 2 Nightingale Breast Screening Centre & Prevent Breast Cancer Unit, Wythenshawe Hospital, Manchester University NHS Foundation Trust, Manchester M23 9LT, UK; Sue.astley@manchester.ac.uk (S.A.); Gareth.Evans@mft.nhs.uk (D.G.E.); anthony.howell@manchester.ac.uk (A.H.); 3 Manchester Breast Centre, The Christie Hospital, Manchester M23 9LT, UK; 4 Division of Informatics, Imaging & Data Sciences, School of Health Sciences, Faculty of Biology, Medicine and Health, University of Manchester, Manchester M13 9PT, UK; 5 Manchester Centre for Genomic Medicine, Manchester University Hospitals NHS Foundation Trust, Manchester M23 9LT, UK; 6 Manchester Centre for Genomic Medicine, NW Genomic Laboratory Hub, Manchester University Hospitals NHS Foundation Trust, Manchester M13 9WL, UK; 7 Manchester Academic Health Science Centre, Division of Evolution and Genomic Sciences, Faculty of Biology, School of Biological Sciences, Medicine and Health, University of Manchester, Manchester M23 9LT, UK; 8 Cambridge Breast Unit, Addenbrooke’s Hospital, Cambridge CB2 0QQ, UK; rmlw2@cam.ac.uk; 9 Girton College, University of Cambridge, Cambridge CB3 0JG, UK; 10 Manchester Academic Health Science Centre, Division of Cancer Sciences, Medicine and Health, University of Manchester, Manchester M23 9LT, UK

**Keywords:** mammographic density, body mass index, weight loss, breast cancer risk, breast cancer prevention, premenopausal

## Abstract

**Simple Summary:**

This study assessed the association between short-term weight change and mammographic density in premenopausal women losing weight through diet and exercise to reduce their risk of postmenopausal breast cancer. We aimed to understand whether a reduction in body mass index affects various components of the breast, which could indicate a potential pathway for the reduction in postmenopausal breast cancer risk seen with premenopausal weight loss. Understanding this pathway is useful for monitoring the effectiveness of prevention strategies based on lifestyle advice. We found that a short-term reduction in premenopausal body mass index through diet and exercise is associated with a reduction in breast fat, but it is unlikely to have a significant effect on the quantity of breast glandular tissue. Breast cancer risk determined by changes in breast density might not capture potential weight loss-induced breast cancer risk reduction, instead falsely ascribing an increased risk due to increased percent density.

**Abstract:**

We evaluated the association between short-term change in body mass index (BMI) and breast density during a 1 year weight-loss intervention (Manchester, UK). We included 65 premenopausal women (35–45 years, ≥7 kg adult weight gain, family history of breast cancer). BMI and breast density (semi-automated area-based, automated volume-based) were measured at baseline, 1 year, and 2 years after study entry (1 year post intervention). Cross-sectional (between-women) and short-term change (within-women) associations between BMI and breast density were measured using repeated-measures correlation coefficients and multivariable linear mixed models. BMI was positively correlated with dense volume between-women (*r* = 0.41, 95%CI: 0.17, 0.61), but less so within-women (*r* = 0.08, 95%CI: −0.16, 0.28). There was little association with dense area (between-women *r* = −0.12, 95%CI: −0.38, 0.16; within-women *r* = 0.01, 95%CI: −0.24, 0.25). BMI and breast fat were positively correlated (volume: between *r* = 0.77, 95%CI: 0.69, 0.84, within *r* = 0.58, 95%CI: 0.36, 0.75; area: between *r* = 0.74, 95%CI: 0.63, 0.82, within *r* = 0.45, 95%CI: 0.23, 0.63). Multivariable models reported similar associations. Exploratory analysis suggested associations between BMI gain from 20 years and density measures (standard deviation change per +5 kg/m^2^ BMI: dense area: +0.61 (95%CI: 0.12, 1.09); fat volume: −0.31 (95%CI: −0.62, 0.00)). Short-term BMI change is likely to be positively associated with breast fat, but we found little association with dense tissue, although power was limited by small sample size.

## 1. Introduction

Mammographic density (herein referred to as ‘density’) is an established risk factor for breast cancer. Women in the highest density category are at a 4- to 6-fold increased risk of breast cancer relative to those with little or no dense tissue [1]. When assessed by mammography, the breast is broadly characterised by two components: fibroglandular dense tissue and fatty non-dense tissue. Percent breast density is measured as the relative proportion of dense tissue in the breast, either in terms of area or volume depending on the measurement method. Visual assessment measures percent density with respect to the total breast area (TA) whilst automated and semi-automated methods can also measure the extent of dense and fatty tissue separately. Both absolute dense area (DA) and percentage dense area (PDA) are positively associated with risk of premenopausal (and postmenopausal) breast cancer [2,3,4], and absolute dense volume (DV) and percentage dense volume (PDV) have also shown positive associations [5,6]. Associations of breast fat area (FA) and volume (FV) with breast cancer risk are unclear, although there is some suggestion of an inverse relationship with premenopausal breast cancer risk [4,6].

In postmenopausal women, higher attained body mass index (BMI) is associated with a higher risk of breast cancer [7,8,9], with an estimated 40% increase in risk for every 10 kg/m^2^ of BMI in never users of hormone replacement therapy [9]. This increase in risk is partly explained by increased aromatisation of androgens to oestrogen in peripheral adipose tissue, which promotes cell proliferation [10,11], carcinogenesis [10,11], and insulin resistance [12]. Whilst BMI is a widely accepted risk factor for breast cancer in postmenopausal women, there may be an inverse relationship in premenopausal women [13].

Weight gain across the premenopausal years has also been linked to an increased risk of postmenopausal breast cancer. Every 5 kg of adult weight gain is associated with an approximate 10% increase in risk amongst never or low-hormone replacement therapy users [14,15]. However, a number of studies (as summarised by Hardefeldt et al. [16]) suggest that these effects are reversible with efficient weight loss [16]. In particular, weight loss in the premenopausal years has been shown to reduce postmenopausal breast cancer risk [17,18]. Risk reductions of approximately 40% have also been seen with large weight losses as a result of bariatric surgery in populations of pre- and postmenopausal women [19].

The effects of short-term weight change on breast density are less well understood, particularly those as a result of dietary weight loss. Mammographic density is a dynamic phenotype and has the potential to respond to short-term weight changes, making density reduction a possible biomarker for reduction in risk as a result of weight loss. This study aims to explore the effect of short-term dietary weight change on density using both area-based and volumetric methods in a cohort of premenopausal women to ascertain whether the relationship between weight loss and reduced postmenopausal breast cancer risk could, in part, be mediated by reductions in mammographic tissue.

## 2. Materials and Methods

### 2.1. Study Design and Participants

The Lifestyle Study is a prospective non-randomised 1 year diet and exercise weight loss intervention study amongst 79 high-risk premenopausal women attending annual screening within the Breast Cancer Family History clinic at the Prevent Breast Cancer research unit at the Manchester University Hospital Foundation NHS Trust [20,21,22,23]. Attendees of our regional Family History Clinic, aged 35–45 years, received a mailed invitation to enter either a 12-month intensive diet and exercise weight loss programme or a usual care group receiving standard written advice only, depending on their proximity to the hospital. Eligibility required women to be premenopausal with regular menstrual cycles, non-smokers, have a self-reported adult weight gain ≥ 7 kg, and a sedentary lifestyle (<40 min moderate physical activity per week). All women had a family history of breast cancer (with lifetime risk 17–40% as assessed by the Tyrer–Cuzick model [24,25]), but were excluded if they had a known *BRCA1/2* mutation or a previous history of cancer. Women were also excluded if they were already successfully dieting or losing weight, were pregnant or planning to become pregnant over the next year, had used hormonal oral contraceptives in the last six months, or had psychiatric or physical co-morbidities that could affect their ability to take part in a diet and physical activity weight loss programme.

In the intervention group (*n* = 40), women followed a 12-month intensive supervised weight loss programme involving a 25% energy-restricted Mediterranean type diet and an individualised physical activity program (150 min moderate intensity physical activity and 40 min of resistance exercise per week). The usual care group (*n* = 39) received standard written advice about diet and physical activity but no additional support for weight loss. Women provided baseline information on alcohol intake (from a 4-day food diary) and physical activity (7-day recall from an interview questionnaire) at their baseline clinic visit. All subjects gave their informed consent for inclusion before they participated in the study. The study was conducted in accordance with the Declaration of Helsinki, and the protocol was approved by the South Manchester Ethics Committee (Reference no. 01/426).

The objective of this analysis was to assess the relationship between BMI and breast density in the entire cohort of women. All participants had changing BMI measures irrespective of the type of weight loss advice they received, hence the intervention and usual care groups were combined and treated as one cohort. Furthermore, to limit the effect of women contributing observations to an area-based measure or volumetric measure only, the cohort was restricted to those with both an area and volumetric density measurement at any one or more time points (*n* = 65, 82% of the cohort).

### 2.2. Mammographic Density

Mammographic films were digitised using a Kodak LS85 digitiser at a pixel size of 50 µm and with 12-bits (4096 grey levels) pixel depth. The images were then anonymised and randomised to ensure the radiologists remained unaware of the time point of each mammogram. Mammograms were analysed using three different methods: (1) a semi-automated area-based measure based on computer-assisted thresholding by a single expert user (Cumulus, Sunnybrook health sciences centre, Toronto, Canada, [26]); (2) an automated volumetric Stepwedge method developed at Manchester University [27]; and (3) a visual assessment score of percentage density read to the nearest 5% by two experienced readers and expressed as an average of the two scores to calculate PDA. Cumulus was used to calculate TA, DA, FA, and PDA, and the Manchester Stepwedge method calculated total volume (TV), DV, FV, and PDV. Density assessments were made at 3 time points: baseline, 1 year follow-up (at the end of the intervention) and 1 year after the end of the intervention. Baseline mammograms were taken at the point of entry to the study; for those women with a mammogram performed within one year of entry, their most recent mammogram within the last 12 months was used. Each woman had four mammographic views taken at each time point: Left Cranial-Caudal, Right Cranial-Caudal, Left Mediolateral-Oblique, and Right Mediolateral-Oblique, and a final mammographic score at each time point was calculated using an average of the four views. The main analysis refers to Cumulus measured area-based density and Stepwedge measured volumetric density only to assess the effects of BMI on dense and non-dense tissue separately. Visually-assessed density had similar results to Cumulus-assessed PDA, so was included as a secondary density measure only. Results for TA and TV are also reported as secondary density measures in the Supplementary Materials.

### 2.3. Body Weight and Body Composition

Weight, BMI, and a variety of different measures of body composition were assessed at baseline, 1 year follow-up (at the end of the intervention), and 1 year after the end of the intervention. Weight (kg) and height (m) were determined using a calibrated beam balance and stadiometer and used to calculate BMI (kg/m^2^). Other body composition assessments were also made such as waist circumference; total body fat, fat free mass and % body fat (assessed using a DXA whole body scanner (Hologic Inc., Bedford, MA, USA) and bioelectrical impedance (Tanita TBF-300A, Tanita Europe B.V., Hoogoorddreef 56E, 1101 BE Amsterdam, The Netherlands)); and intra-abdominal and abdominal subcutaneous area (assessed using a magnetic resonance imaging (MRI) scan with a single transverse scan taken at the level of the intervertebral disc between the L2 and L3 vertebrae). Weight, BMI, waist circumference, and total body fat, fat free mass, and % body fat (impedance) were recorded at all three time points. Intra-abdominal area, abdominal subcutaneous area, and total body fat, fat free mass, and % body fat (DXA) were only measured at baseline and at 1 year. Weight at age 20 years was self-reported via questionnaire, and BMI at age 20 years was calculated using weight at age 20 years and height at study entry. Long-term adult BMI gain was calculated as the difference between baseline BMI and BMI at age 20 years. We discuss BMI as the measure of body weight throughout the main analysis because BMI is a commonly used adjustment for density and it is a well-established risk factor for breast cancer. Other body composition measures gave similar correlations with density to those of BMI and were highly correlated with BMI. Therefore, other body composition measures are included as secondary analyses in the Supplementary Materials. Weight gain during the intervention was defined as ≥+3% of baseline weight, weight loss was defined as ≤−3% of baseline weight, and a weight change >−3% to <+3% of the baseline weight was defined as a stable weight [28].

### 2.4. Statistical Analysis

Data were visualised using custom-made ‘tadpole plots’, where each tadpole represents a woman, the head plots the woman’s BMI and density at her last time point, and the points on the tail plot her BMI and density at earlier time points. Correlation (*r*) between BMI and mammographic density was assessed on a cross-sectional basis (between women), and within women as their short-term BMI changed, using repeated measures methods that use all of the measurements at the same time [29,30]. Briefly, between women correlation was a weighted Pearson correlation coefficient [30], and within women correlation was based on the decomposition of sums of squares from an analysis of variance [29]. The 95% confidence intervals were estimated using an empirical bootstrap (10,000 resamples). The simultaneous association of between and within women correlations was tested using a linear mixed model adjusted for age [31] (Appendix A). To help with comparisons across different measures of breast density, the breast density values were first standardised (Appendix B). To make density measures more symmetric and approximately normally-distributed, they were transformed: a square root transformation for area measures and a cube root transformation for volumetric measures. An exploratory analysis was undertaken to assess the effect of adding BMI gain since 20 years of age to the model. An additional exploratory analysis tested whether there was an association between breast density and DXA bone density. A sensitivity analysis assessed repeated measures correlation coefficients for BMI and density stratified by intervention group.

Analysis used the statistical software R [32]. All tests were two-sided and considered significant at the 5% level.

## 3. Results

Baseline characteristics of the cohort are shown in Table 1. Median age was 41 years (interquartile range (IQR), 38–43 years), and the majority of women were Caucasian (*n* = 60, 92%) and parous (*n* = 55, 85%). At baseline, 27 women (42%) were classified as overweight (BMI ≥ 25 kg/m^2^ and <30 kg/m^2^), 20 (31%) were obese (BMI ≥ 30 kg/m^2^), and 18 (28%) were in the normal BMI range (BMI ≥ 18.5 kg/m^2^ and <25 kg/m^2^). By the end of the 2 year study period (1 year post intervention), 22 women (34%) had lost weight, 16 (25%) had gained weight, and 26 (41%) maintained their original weight. Overall, women in the intervention group lost more weight than the usual care group (mean percentage of baseline weight at 1 year = −4.4% and 0.1%, respectively; mean percentage of baseline weight at 2 years = −2.9% and 2.0%, respectively).

Median PDA, DA, and FA of each woman’s average density measure over the intervention were 37.1% (IQR, 2.5%–71.3%), 59.9 cm^2^ (IQR, 5.8–158.4 cm^2^) and 107.3 cm^2^ (IQR, 23.6–405.1 cm^2^), respectively. For Stepwedge measures, PDV, DV, and FV were 22.7% (IQR, 6.7%–69.4%), 191.5 cm^3^ (IQR, 56.7–710.4 cm^3^), and 573.0 cm^3^ (IQR, 72.8–1992.1 cm^3^), respectively. A flow chart detailing the availability of mammographic density measures across the intervention is shown in Figure S1 (all women had BMI available at all time-points except for one woman with missing BMI at 2 years—this data point was excluded from analyses involving BMI).

**Figure 1:**
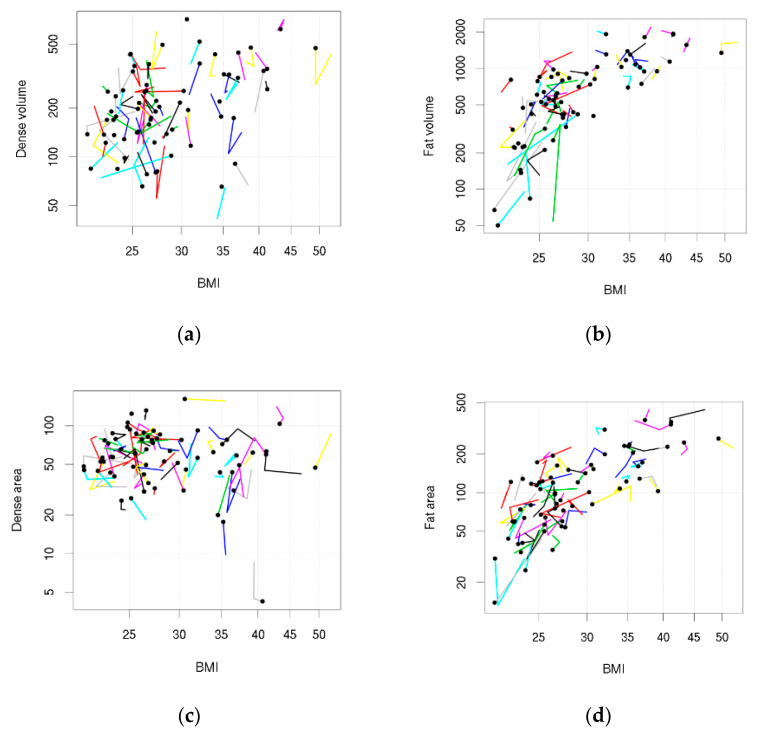
Tadpole plots showing body mass index (BMI) and density measures across the 2 year follow-up. Each tadpole represents a woman: the tadpole head shows BMI and density (if density is available) at her last follow-up and the points on the tail show BMI and density (if density is available) at her earlier follow-ups. (**a**) Dense volume; (**b**) Fat volume; (**c**) Dense area; (**d**) Fat area.

Table 2 shows the repeated measures correlations. DV was positively correlated with BMI between women (*r* = 0.41, 95%CI 0.17 to 0.61) but less so within women (*r* = 0.08, 95%CI −0.16 to 0.28). There was little association between DA and BMI (between women *r* = −0.12, 95%CI −0.38 to 0.16; within women *r* = 0.01, 95%CI −0.24 to 0.25). PDV was inversely associated with BMI between and within women (between *r* = −0.48, 95%CI −0.64 to −0.33; within *r* = −0.36, 95%CI −0.54 to −0.12), and PDA was inversely associated with BMI between women (*r* = −0.58, 95%CI −0.72 to −0.42), but less so within women (*r* = −0.22, 95%CI −0.44 to 0.01). FV and FA were positively correlated with BMI between and within women (volume: between *r* = 0.77, 95%CI 0.69 to 0.84, within *r* = 0.58, 95%CI 0.36 to 0.75; area: between *r* = 0.74, 95%CI 0.63 to 0.82, within *r* = 0.45, 95%CI 0.23 to 0.63). The magnitude of correlations was stronger between women than within women. These associations were also seen in Figure 1 when data were visually assessed using tadpole plots (trends in the tadpole heads represented the between women correlations and trends in the tadpole tails represented within women correlations).

Results for repeated measures correlation coefficients were similar when evaluated in a sensitivity analysis stratifying the cohort by intervention group. Within women associations for BMI and FA or FV were slightly stronger for women following the supervised weight loss programme compared with the usual care group, but there was little association (within women) for BMI and DA or DV in both intervention groups (Table S6).

Other body fat composition measures were highly correlated with BMI (Table S3), and the associations between breast density and other body fat compositions were similar to those with BMI (Tables S1 and S2). The correlations between various mammographic density measures are also reported in the Supplementary Materials (Table S4).

The between and within women associations for density and BMI measures were similar when estimated jointly in an age-adjusted linear mixed model (Table 3). In a sensitivity analysis, the same model was fit using weight instead of BMI, but it had a worse model fit for almost all density measures (Table S5).

When a term for BMI gain since age 20 years was added to the linear mixed model, the model fit improved for PDA, PDV, FV, and DA (all ΔLR-χ^2^ *p* < 0.05) (Table 4). After including BMI gain since age 20 years, between women associations for BMI became more strongly inversely associated with percent density (approximately −0.5 to −0.8), more strongly positively associated with breast fat (approximately 0.6 to 0.8), more strongly inversely associated with DA (−0.1 to −0.5), and less strongly positively associated with DV (0.4 to 0.2). Within women effects of BMI on density were almost unchanged when including BMI gain since age 20 years. BMI gain from age 20 years (adjusted for attained BMI) was positively associated with DA, PDA, and PDV (5 kg/m^2^ increase in BMI gain since age 20 years was associated with 0.61 (95%CI 0.12 to 1.09), 0.61 (95%CI 0.21 to 1.02), and 0.47 (95%CI 0.05 to 0.88) standard deviation increase in breast density (β), respectively), and inversely associated with FV (*β* = −0.31, 95%CI −0.62 to 0.00), but less association was seen with DV (*β* = 0.15, 95%CI −0.29 to 0.59) and FA (*β* = −0.32, 95%CI −0.67 to 0.03). 

Finally, in tests of association between breast and bone density, there was some indication of a positive between women correlation for bone density and FV (*r* = 0.26, 95%CI, 0.00 to 0.50), DV (*r* = 0.33, 95%CI, 0.09 to 0.54), and TV (*r* = 0.31, 95%CI, 0.06 to 0.54) (Table S1), but we found little correlation within women (Table S2).

## 4. Discussion

The data in this study provide some support for the two main findings. First, it is possible that the higher a premenopausal woman’s BMI, the higher her breast fat and dense tissue (in particular, dense volume), and the lower her percent density. Second, the data suggested that as a premenopausal woman loses weight, her breast fat reduces, dense tissue remains relatively unchanged, and percent dense tissue increases. Effective weight loss during premenopausal years has been associated with a reduced risk of postmenopausal breast cancer [16,17,18], but our study data suggest that risk reduction is unlikely to be mediated by a short-term reduction in dense breast tissue. This is likely to mean that incorporation of change in percent breast density into risk algorithms will not capture potential weight loss-induced breast cancer risk reduction and may falsely ascribe an increased risk due to increased percent density. Therefore, risk prediction models need to consider how best to incorporate changes in weight and mammographic density when predicting breast cancer risk.

The between women associations of attained premenopausal BMI and breast density observed in this study were consistent with previous studies. High BMI is associated with high dense volume [33,34,35], but the correlation between BMI and dense area is less strong, and often close to zero [36,37,38,39]. These differences are likely to be a result of volumetric measures representing breast tissue more accurately than area-based methods by accounting for breast thickness and overlapping tissue. Additionally, since the breast is a deposit for adipose tissue, high attained BMI is strongly associated with high levels of breast fat area [36,37,38,39] and breast fat volume [33,34], which in turn leads to an inverse association between BMI and both percent dense area [36,37,38,39,40,41] and percent dense volume [33,34,35,42,43].

There have been very few studies to assess the effect of dietary weight loss on breast density in premenopausal women. Boyd et al. reported reductions in total and dense area alongside modest weight change within an intervention trial of women on a 2-year low-fat, high-carbohydrate diet [44]. In particular, a 5.4% decrease in dense area was seen in premenopausal women in the low-fat diet group with a 0.1kg/m^2^ BMI reduction (*n* = 249) compared with a 2.5% decrease in the control group with a 0.3kg/m^2^ BMI gain (*n* = 264). These reductions may be associated with the large reductions in dietary fat (55 to 35 g/day) and saturated fat (19 to 12 g/day) rather than weight loss in this study. This was considerably higher than those advised and achieved in the current reported study (total fat reduced from 77 to 60 g/day and saturated fat reduced from 28 to 21 g/day). Other trials have also assessed the effect of lifestyle interventions for weight loss on breast density, although in postmenopausal women only. In the ALPHA trial, postmenopausal women on a 1-year aerobic exercise intervention lost on average 39 cm^3^ more breast fat than the controls, but there was little difference in the change in dense tissue between the two groups [45]. Furthermore, the DAMA trial reported a reduction in volumetric percent density of approximately 14% for postmenopausal women following a 2-year diet or exercise intervention when compared with the controls [46]. Large weight loss with bariatric surgery is also associated with large reductions in breast fat alongside relatively smaller reductions in dense tissue, and an increase in percent density [47,48,49].

As an exploratory analysis, we also found an association between increased BMI gain since 20 years of age and higher dense tissue and percent density. It is possible that this is a pathway for the increased risk of postmenopausal breast cancer seen with adult weight gain [7,15,50,51,52]. However, this association is likely to reflect the inverse association seen in previous studies between adolescent body adiposity and dense tissue in later life [38,40,53,54,55], since, given the adjustment for current BMI, women with greater gain in BMI will have had lower BMI at 20 years of age. This interesting observation requires further investigation in larger datasets of women. Additionally, exploratory analysis of bone density found little association with breast density, which is in agreement with previous studies [56].

Strengths of this study include the various measures of breast density including Cumulus and the Stepwedge method, which allowed for the assessment of dense and fatty tissue separately as well as various measures of body weight to assess adiposity. The study also assessed breast density as an area-based measure and volumetrically; both of which have similar abilities for breast cancer risk prediction [57]. Additionally, all women were encouraged to lose weight, which produced data with large within women variation in BMI, in turn increasing the potential to see an effect of changing BMI on mammographic density. Furthermore, the Lifestyle Study provided a data source to assess premenopausal weight loss and density associations; something that is not possible in studies involving routine screening data. This also provided a greater ability to capture the effects of weight loss on density because this cohort of premenopausal women were likely to have had higher dense tissue at baseline (with greater ability to decrease) than screening populations involving postmenopausal women [58]. Finally, the use of repeated measures over a 2-year period allowed us to assess the association between BMI and breast density longitudinally, whilst making use of all available data simultaneously.

Limitations of the study include the small sample size, which limits statistical power. This is particularly relevant for volumetric measures, which had a moderate amount of missing data at the baseline. In addition, the study design was not powered for the analysis of mammographic density, which was a secondary analysis (the study was powered for salivary oestradiol). This was a relatively small study, and ideally, a larger study with sufficient power would be run to verify our evidence. Another limitation is the analysis of BMI gain since 20 years of age relies on self-reported information on weight at age 20 years. This may be less accurate than the measured values. However, validation studies show that self-reported BMI is highly correlated with independently measured BMI, and the mean difference between self-reported and measured weight is minimal [59,60]. Finally, breast thickness is likely to have changed whilst women lost weight during the intervention. Volumetric measures are influenced by breast thickness [61], hence there might have been larger variation in the serial compared with stable volumetric measurements, resulting in reduced ability to capture the within women effects of BMI on dense tissue volumetrically.

## 5. Conclusions

This study suggests that premenopausal weight loss reduces breast fat but that it does not reduce dense tissue. Short-term premenopausal weight loss is likely to be linked to lower postmenopausal breast cancer risk through reductions in adipose tissue, not fibroglandular tissue. This means that a potential breast cancer risk reduction as a result of weight loss might not be captured by changes in breast density, and the resulting increase in percent density may falsely ascribe an increase in risk. However, the study was limited by the small sample size, and more studies are required to provide evidence to confirm these results.

## Figures and Tables

**Table 1:** Participant characteristics at study entry.

Characteristic	All	Intervention	Usual Care
Total	65	33	32
Age * (years)	41 (38–43)	41 (39–43)	40 (38–42)
Baseline BMI * (kg/m^2^)	27.1 (24.7–33.4)	27.1 (25.1–31.9)	27.0 (24.4–34.0)
Baseline BMI categories ^#^ (kg/m^2^)			
Normal (≥18.5 to <25)	18 (28%)	7 (21%)	11 (34%)
Overweight (≥25 to <30)	27 (42%)	16 (48%)	11 (34%)
Obese (≥30)	20 (31%)	10 (30%)	10 (31%)
BMI gain since 20 years * (kg/m^2^)	5.8 (4.7–9.4)	6.3 (4.7–10.0)	5.7 (4.6–8.9)
Height * (m)	1.64 (1.60–1.68)	1.63 (1.60–1.68)	1.65 (1.59–1.68)
Age at menarche * (years)	12 (12–13)	12 (12–13)	12 (12–13)
Number of live births ^#^			
Nulliparous	10 (15%)	6 (18%)	4 (13%)
1–2	41 (63%)	20 (61%)	21 (66%)
3–4	12 (18%)	6 (18%)	6 (19%)
≥5	2 (3%)	1 (3%)	1 (3%)
Age first live birth * (years)	27 (22–29)	27 (24–31)	26 (22–29)
Ethnicity ^#^ (% Caucasian)	60 (92%)	29 (88%)	31 (97%)
Previous smoker ^#^			
Never	54 (83%)	29 (88%)	25 (78%)
Ever	11 (17%)	4 (12%)	7 (22%)
Previous oral contraception use ^#^			
Never	5 (8%)	3 (9%)	2 (6%)
Ever	58 (89%)	29 (88%)	29 (91%)
Unknown	2 (3%)	1 (3%)	1 (3%)
Breastfed ^#^			
Never	22 (34%)	12 (36%)	10 (31%)
Ever	41 (63%)	21 (64%)	20 (63%)
Unknown	2 (3%)	0 (0%)	2 (6%)
10-year Tyrer–Cuzick risk * (%)	4 (3–5)	5 (4–6)	3 (3–4)
Alcohol intake ^a,^* (units/week)	11 (3–24)	11 (3–22)	10 (1.5–26)
Physical activity ^b,^* ((kJ/kg)/week)	974 (945–999)	968 (941–999)	978 (953–1007)
Weight change from baseline to 1 year, categories ^#^			
Loss	26 (40%)	20 (61%)	6 (19%)
Stable	27 (42%)	9 (27%)	18 (56%)
Gain	12 (18%)	4 (12%)	8 (25%)
Weight change from baseline to 1 year **	−2.2 (5.4)	−4.4 (5.0)	0.1 (4.8)
Weight change from baseline to 2 years, categories ^#^			
Loss	22 (34%)	16 (48%)	6 (19%)
Stable	26 (41%)	13 (39%)	13 (42%)
Gain	16 (25%)	4 (12%)	12 (39%)
Weight change from baseline to 2 years **	−0.5 (7.1)	−2.9 (6.2)	2.0 (7.1)

BMI: Body mass index. ^#^ N (%); * Median (interquartile range); ** Mean (standard deviation) % of baseline weight (kg). ^a^ Alcohol from a 4-day food diary; ^b^ Physical activity from 7-day recall. Weight loss defined as ≤−3% of baseline weight (kg); Stable weight defined as >−3% to <+3% of baseline weight (kg); Weight gain defined as ≥+3% of baseline weight (kg).

**Table 2:** Repeated measures between women and within women correlations for mammographic density and body mass index.

Field	VAS (95%CI) (sqrt%)	PDA (95%CI) (sqrt%)	PDV (95%CI) (cbrt%)	FA (95%CI) (sqrt)	FV (95%CI) (cbrt)	DA (95%CI) (sqrt)	DV (95%CI) (cbrt)
Cross-sectional BMI (between women)	−0.62 (−0.74 to −0.47)	−0.58 (−0.72 to −0.42)	−0.48 (−0.64 to −0.33)	0.74 (0.63 to 0.82)	0.77 (0.69 to 0.84)	−0.12 (−0.38 to 0.16)	0.41 (0.17 to 0.61)
Short-term BMI change (within women)	−0.27 (−0.48 to −0.05)	−0.22 (−0.44 to 0.01)	−0.36 (−0.54 to −0.12)	0.45 (0.23 to 0.63)	0.58 (0.36 to 0.75)	0.01 (−0.24 to 0.25)	0.08 (−0.16 to 0.28)

VAS: Visual assessment score; PDA: percent dense area; PDV: percent dense volume; FA: fat area; FV: fat volume; DA: dense area; DV: dense volume; sqrt: square root transformed; cbrt: cube root transformed; BMI: body mass index; 95%CI: 95% confidence interval. Area-based measures from Cumulus; volumetric measures from Manchester Stepwedge. Within women correlations represent trends over the entire 2 year period.

**Table 3:** Multivariable linear mixed model fit results for mammographic density on body mass index (between and within women), adjusted for age (A1).

Density Outcome	Intercept (95%CI)	Age (95%CI) (Per 10 Years)	BMI (95%CI) [between] (Per 5 kg/m^2^)	BMI (95%CI) [within] (Per 5 kg/m^2^)
VAS (sqrt%)	3.75 (1.88 to 5.61)	−0.19 (−0.56 to 0.19)	−0.51 (−0.68 to −0.35)	−0.27 (−0.44 to −0.10)
PDA (sqrt%)	2.87 (0.57 to 5.17)	−0.05 (−0.53 to 0.43)	−0.46 (−0.63 to −0.30)	−0.32 (−0.59 to −0.05)
PDV (cbrt%)	1.73 (−1.07 to 4.53)	0.12 (−0.48 to 0.71)	−0.39 (−0.57 to −0.21)	−0.85 (−1.32 to −0.39)
FA (sqrt)	−3.63 (−5.25 to −2.02)	0.04 (−0.28 to 0.36)	0.60 (0.46 to 0.74)	0.43 (0.27 to 0.58)
FV (cbrt)	−3.46 (−5.27 to −1.64)	−0.04 (−0.42 to 0.34)	0.63 (0.50 to 0.76)	0.79 (0.56 to 1.03)
DA (sqrt)	0.57 (−2.13 to 3.27)	−0.03 (−0.59 to 0.53)	−0.08 (−0.28 to 0.11)	0.01 (−0.30 to 0.33)
DV (cbrt)	−2.39 (−5.11 to 0.33)	0.09 (−0.48 to 0.66)	0.35 (0.16 to 0.53)	0.16 (−0.24 to 0.55)

VAS: Visual assessment score; PDA: percent dense area; PDV: percent dense volume; FA: fat area; FV: fat volume; DA: dense area; DV: dense volume; sqrt: square root transformed; cbrt: cube root transformed; BMI: body mass index; 95%CI: 95% confidence interval. Area-based measures from Cumulus; volumetric measures from Manchester Stepwedge. Between women BMI calculated as the mean BMI for each woman; within women BMI calculated as the difference between each woman’s BMI and her mean BMI. Density measures are standardised (see Appendix B). One woman with missing BMI at age 20 years excluded. Within women effects represent trends over the entire 2 year period.

**Table 4:** Multivariable linear mixed model fit results for mammographic density on body mass index (between and within women) and body mass index gain since 20 years of age, adjusted for age (A2).

Density Outcome	Intercept (95%CI)	Age (95%CI) (Per 10 Years)	BMI (95%CI) (between) (Per 5kg/m^2^)	BMI (95%CI) (within) (Per 5kg/m^2^)	BMI Gain Since 20 Years of Age (95%CI) (Per 5kg/m^2^)	ΔLR-χ^2^ *p*-Value (A1 vs. A2)
VAS (sqrt%)	5.47 (3.34 to 7.60)	−0.25 (−0.61 to 0.12)	−0.92 (−1.23 to −0.62)	−0.27 (−0.45 to −0.10)	0.59 (0.20 to 0.97)	0.0031
PDA (sqrt%)	4.90 (2.34 to 7.46)	−0.16 (−0.63 to 0.31)	−0.89 (−1.22 to −0.57)	−0.32 (−0.59 to −0.06)	0.61 (0.21 to 1.02)	0.0033
PDV (cbrt%)	3.35 (0.30 to 6.40)	0.01 (−0.57 to 0.60)	−0.71 (−1.05 to −0.38)	−0.85 (−1.32 to −0.39)	0.47 (0.05 to 0.88)	0.0267
FA (sqrt)	−4.59 (−6.49 to −2.69)	0.08 (−0.24 to 0.40)	0.82 (0.54 to 1.10)	0.43 (0.28 to 0.59)	−0.32 (−0.67 to 0.03)	0.0704
FV (cbrt)	−4.42 (−6.44 to −2.40)	0.01 (−0.37 to 0.38)	0.84 (0.59 to 1.09)	0.79 (0.56 to 1.03)	−0.31 (−0.62 to 0.00)	0.0476
DA (sqrt)	2.58 (−0.48 to 5.64)	−0.14 (−0.70 to 0.41)	−0.51 (−0.90 to −0.12)	0.01 (−0.31 to 0.32)	0.61 (0.12 to 1.09)	0.0145
DV (cbrt)	−1.90 (−4.96 to 1.15)	0.06 (−0.51 to 0.64)	0.24 (−0.12 to 0.60)	0.16 (−0.24 to 0.55)	0.15 (−0.29 to 0.59)	0.4967

VAS: Visual assessment score; PDA: percent dense area; PDV: percent dense volume; FA: fat area; FV: fat volume; DA: dense area; DV: dense volume; sqrt: square root transformed; cbrt: cube root transformed; BMI: body mass index; 95%CI: 95% confidence interval. Area-based measures from Cumulus; volumetric measures from Manchester Stepwedge. Between women BMI calculated as the mean BMI for each woman; within women BMI calculated as the difference between each woman’s BMI and her mean BMI; BMI gain from age 20 years calculated as the difference between each woman’s BMI at baseline and her BMI at age 20 years. Density measures are standardised (see Appendix B). One woman with missing BMI at age 20 years excluded. ΔLR-χ^2^ represents the difference in likelihood ratio for A1 versus A2. Within women effects represent trends over the entire 2 year period. All variables adjusted for each other in the multivariable model, therefore BMI gain since 20 years of age is adjusted for current BMI through the variable for between women BMI.

## Data Availability

The dataset used and analysed during the current study is available from the corresponding author on reasonable request.

## Supplementary Materials

The following are available online at https://www.mdpi.com/article/10.3390/cancers13133245/s1, Table S1: Complete results for repeated measures between women correlations for mammographic density and body composition measures; Table S2: Complete results for repeated measures within women correlations for mammographic density and body composition measures; Table S3: Complete results for repeated measures between women correlations for different body composition measures; Table S4: Complete results for repeated measures between women correlations for different mammographic density measures; Table S5: Multivariable linear mixed model fit results for A1 using either body mass index or weight; Table S6: Repeated measures between women and within women correlations for mammographic density and body mass index, stratified by intervention group; Figure S1: Flow chart of women included in the analysis and availability of mammographic density data.

## Abbreviations

DA	dense area
PDA	percent dense area
FA	fat area
TA	total area
DV	dense volume
PDV	percent dense volume
FV	fat volume
TV	total volume
BMI	body mass index
VAS	visual assessment score

## Appendix A

Linear mixed model for mammographic density on body mass index and age. A linear mixed model was used to model density and body mass index (BMI) associations in Table 3. This model allows for repeated measures and uses all of the available data (missing pairs of density and BMI are excluded). Breast density $y_{ij}$ for woman i = 1, …, *n* at time j = {1,2,3} is modelled as:

(A1) $$y_{ij}=\alpha +\beta age_{ij}+\gamma {\bar{x}}_{i.}+\delta \left(x_{ij}-{\bar{x}}_{i.}\right)+u_{i}+e_{ij};$$

where α is an overall intercept; $age_{ij}$ is the age at baseline for woman i at time j; β is the slope for age; ${\bar{x}}_{i.}$ is mean BMI for woman i; γ is the between women slope; $x_{ij}$ is the BMI of woman i at time j; δ is the within women slope; and $e_{ij}$ is an independent random error. Another term that allows for differences between women in their overall density level is the independent random intercept $u_{i}$ for woman i. The model is completed by assuming normal distributions for $u_{i}$ and $e_{ij}$ with zero mean, unknown variances, and zero covariance. The model was fitted by maximum likelihood. To aid interpretation of the estimates across different measures of density, the density values were standardised (see Appendix B). To test γ = 0 (between women correlation) and δ = 0 (within women correlation), a Wald test was applied.

The model was extended to consider BMI gain from age 20 years in Table 4:

(A2) $$y_{ij}=\alpha +\beta age_{ij}+\gamma {\bar{x}}_{i.}+\delta \left(x_{ij}-{\bar{x}}_{i.}\right)+u_{i}+\varepsilon z_{i}+e_{ij};$$

where $z_{i}$ is the BMI gain since age 20 years for woman i: calculated as the difference between baseline BMI for woman i and BMI at age 20 years for woman i, and ε is the slope for BMI gain since age 20 years. To test ε = 0, a Wald test was applied.

## Appendix B

Standardisation of each mammographic density measure:

$$\bar{x}=\frac{\sum_{i=1}^{n_{\;}}{\bar{x}}_{i}}{n}$$

$$\sigma =\sqrt{\frac{\sum_{i=1}^{n}{\left({\bar{x}}_{i}-\bar{x}\right)}^{2}}{n-1}}$$

$$z_{ij}=\frac{d_{ij}-\bar{x}\;}{\sigma }$$

where ${\bar{x}}_{i}$ is the mean density for woman i = 1, …, *n*; $d_{ij}$ is the density measure for woman i = 1, …, *n* at time point j = {1,2,3}; and $z_{ij}$ is the standardised density measure for woman i at time point j.
