# Supplementary material for: The Relationship between Body Mass Index and Mammographic Density during a Premenopausal Weight Loss Intervention Study
Source: Cancers (Basel). 2021 Jun 29;13(13):3245. doi: 10.3390/cancers13133245 (PMC8269424; doi:10.3390/cancers13133245)
Supplement: Supplementary file 1 [file cancers-13-03245-s001.zip › cancers-1194930-supplementary.pdf]

cancers MDPI

Supplementary

The relationship between body mass index and mammographic density during a premenopausal weight loss intervention study

Emma C. Atakpa, Adam R. Brentnall, Susan Astley, Jack Cuzick, D. Gareth Evans, Ruth M.L. Warren, Anthony Howell, Michelle Harvie*

Table S1. Complete results for repeated measures between women correlations for mammographic density and body composition measures.

Body Composition Measure	VAS (95%CI) (sqrt%)	PDA (95%CI) (sqrt%)	PDV (95%CI) (cbrt%)	FA (95%CI) (sqrt)	DA (95%CI) (sqrt)	TA (95%CI) (sqrt)	FV (95%CI) (cbrt)	DV (95%CI) (cbrt)	TV (95%CI) (cbrt)
Weight	-0.55 (-0.70 to -0.37)	-0.49 (-0.64 to -0.30)	-0.40 (-0.56 to -0.23)	0.69 (0.59 to 0.79)	0.00 (-0.28 to 0.27)	0.71 (0.61 to 0.80)	0.74 (0.65 to 0.82)	0.49 (0.28 to 0.66)	0.77 (0.69 to 0.85)
BMI	-0.62 (-0.74 to -0.47)	-0.58 (-0.72 to -0.42)	-0.48 (-0.64 to -0.33)	0.74 (0.63 to 0.82)	-0.12 (-0.38 to 0.16)	0.72 (0.59 to 0.82)	0.77 (0.69 to 0.84)	0.41 (0.17 to 0.61)	0.78 (0.70 to 0.86)
Waist	-0.63 (-0.76 to -0.46)	-0.59 (-0.73 to -0.41)	-0.54 (-0.67 to -0.38)	0.77 (0.67 to 0.85)	-0.09 (-0.34 to 0.18)	0.76 (0.64 to 0.85)	0.80 (0.72 to 0.87)	0.37 (0.12 to 0.59)	0.79 (0.69 to 0.87)
Imped(total fat)	-0.57 (-0.71 to -0.39)	-0.53 (-0.68 to -0.35)	-0.42 (-0.58 to -0.26)	0.71 (0.61 to 0.80)	-0.03 (-0.32 to 0.25)	0.72 (0.61 to 0.81)	0.76 (0.67 to 0.84)	0.48 (0.26 to 0.66)	0.79 (0.70 to 0.86)
Imped(% fat)	-0.57 (-0.71 to -0.40)	-0.55 (-0.69 to -0.38)	-0.49 (-0.66 to -0.30)	0.71 (0.60 to 0.80)	-0.06 (-0.33 to 0.22)	0.71 (0.59 to 0.81)	0.78 (0.68 to 0.86)	0.43 (0.20 to 0.62)	0.79 (0.69 to 0.87)
Imped(lean)	-0.40 (-0.58 to -0.17)	-0.34 (-0.55 to -0.13)	-0.26 (-0.45 to -0.07)	0.57 (0.44 to 0.69)	0.06 (-0.23 to 0.33)	0.59 (0.47 to 0.71)	0.60 (0.46 to 0.71)	0.45 (0.25 to 0.62)	0.64 (0.51 to 0.74)
DXA(total fat)	-0.55 (-0.69 to -0.36)	-0.53 (-0.68 to -0.35)	-0.54 (-0.68 to -0.39)	0.69 (0.58 to 0.79)	-0.02 (-0.32 to 0.26)	0.69 (0.57 to 0.79)	0.76 (0.68 to 0.84)	0.35 (0.06 to 0.58)	0.77 (0.68 to 0.85)
DXA(lean)	-0.36 (-0.56 to -0.15)	-0.30 (-0.52 to -0.07)	-0.23 (-0.41 to -0.04)	0.53 (0.39 to 0.66)	0.13 (-0.21 to 0.42)	0.59 (0.45 to 0.71)	0.58 (0.45 to 0.70)	0.52 (0.29 to 0.70)	0.64 (0.51 to 0.74)
DXA(% fat)	-0.53 (-0.69 to -0.31)	-0.55 (-0.70 to -0.36)	-0.64 (-0.77 to -0.49)	0.63 (0.48 to 0.76)	-0.09 (-0.35 to 0.17)	0.61 (0.45 to 0.74)	0.72 (0.60 to 0.81)	0.16 (-0.12 to 0.43)	0.69 (0.55 to 0.80)
DXA(bone)	-0.23 (-0.47 to 0.03)	-0.08 (-0.31 to 0.17)	-0.03 (-0.31 to 0.25)	0.17 (-0.08 to 0.41)	0.11 (-0.14 to 0.37)	0.22 (-0.03 to 0.47)	0.26 (0.00 to 0.50)	0.33 (0.09 to 0.54)	0.31 (0.06 to 0.54)
MRI(subcutaneous)	-0.64 (-0.77 to -0.46)	-0.62 (-0.76 to -0.46)	-0.60 (-0.73 to -0.48)	0.74 (0.64 to 0.83)	-0.13 (-0.42 to 0.15)	0.73 (0.62 to 0.81)	0.78 (0.70 to 0.85)	0.25 (-0.04 to 0.48)	0.77 (0.67 to 0.85)
MRI(abdominal)	-0.65 (-0.76 to -0.48)	-0.65 (-0.77 to -0.49)	-0.60 (-0.73 to -0.48)	0.78 (0.68 to 0.86)	-0.15 (-0.42 to 0.10)	0.76 (0.63 to 0.85)	0.80 (0.73 to 0.86)	0.28 (-0.01 to 0.51)	0.79 (0.70 to 0.86)
MRI(total fat)	-0.57 (-0.69 to -0.42)	-0.61 (-0.73 to -0.47)	-0.52 (-0.66 to -0.38)	0.76 (0.61 to 0.86)	-0.18 (-0.39 to 0.05)	0.73 (0.55 to 0.85)	0.75 (0.65 to 0.83)	0.34 (0.07 to 0.54)	0.75 (0.64 to 0.84)

$p < 0.05$; $p > 0.05$; $p = 0.05$. VAS: Visual assessment score; PDA: percent dense area; PDV: percent dense volume; FA: fat area; FV: fat volume; DA: dense area; DV: dense volume; TA: total area; TV: total volume; sqrt: square root transformed; cbrt: cube root transformed; BMI: body mass index; Imped: impedance; 95%CI: 95% confidence interval. Area-based measures from Cumulus; volumetric measures from Manchester Stepwedge.

Table S2. Complete results for repeated measures within women correlations for mammographic density and body composition measures.

Body Composition Measure	VAS (95%CI) (sqrt%)	PDA (95%CI) (sqrt%)	PDV (95%CI) (cbrt%)	FA (95%CI) (sqrt)	DA (95%CI) (sqrt)	TA (95%CI) (sqrt)	FV (95%CI) (cbrt)	DV (95%CI) (cbrt)	TV (95%CI) (cbrt)
Weight	-0.27 (-0.49 to -0.05)	-0.22 (-0.44 to 0.02)	-0.37 (-0.55 to -0.13)	0.46 (0.24 to 0.65)	0.01 (-0.24 to 0.25)	0.54 (0.36 to 0.69)	0.59 (0.37 to 0.76)	0.07 (-0.17 to 0.28)	0.75 (0.55 to 0.85)
BMI	-0.27 (-0.48 to -0.05)	-0.22 (-0.44 to 0.01)	-0.36 (-0.54 to -0.12)	0.45 (0.23 to 0.63)	0.01 (-0.24 to 0.25)	0.54 (0.35 to 0.69)	0.58 (0.36 to 0.75)	0.08 (-0.16 to 0.28)	0.74 (0.54 to 0.85)
Waist	-0.17 (-0.38 to 0.06)	-0.10 (-0.31 to 0.12)	-0.23 (-0.42 to 0.00)	0.25 (0.03 to 0.47)	0.01 (-0.19 to 0.21)	0.32 (0.10 to 0.51)	0.37 (0.09 to 0.58)	0.05 (-0.15 to 0.24)	0.46 (0.14 to 0.68)
Imped(total fat)	-0.22 (-0.44 to 0.03)	-0.24 (-0.46 to 0.01)	-0.32 (-0.52 to -0.09)	0.44 (0.22 to 0.63)	-0.07 (-0.31 to 0.17)	0.47 (0.27 to 0.63)	0.47 (0.20 to 0.67)	0.01 (-0.24 to 0.24)	0.58 (0.29 to 0.76)
Imped(% fat)	-0.14 (-0.35 to 0.10)	-0.28 (-0.47 to -0.03)	-0.23 (-0.45 to 0.03)	0.44 (0.21 to 0.62)	-0.10 (-0.29 to 0.11)	0.43 (0.19 to 0.62)	0.36 (0.05 to 0.60)	0.09 (-0.15 to 0.29)	0.48 (0.15 to 0.70)
Imped(lean)	-0.29 (-0.63 to 0.03)	-0.25 (-0.67 to 0.28)	-0.34 (-0.64 to 0.03)	0.51 (0.05 to 0.81)	-0.12 (-0.57 to 0.40)	0.65 (0.26 to 0.85)	0.52 (0.06 to 0.79)	-0.03 (-0.46 to 0.38)	0.55 (-0.04 to 0.83)
DXA(total fat)	-0.08 (-0.51 to 0.34)	-0.24 (-0.56 to 0.16)	-0.27 (-0.61 to 0.24)	0.46 (0.12 to 0.71)	0.07 (-0.31 to 0.41)	0.55 (0.27 to 0.75)	0.48 (0.03 to 0.77)	0.19 (-0.22 to 0.53)	0.65 (0.15 to 0.87)
DXA(lean)	-0.05 (-0.50 to 0.33)	-0.07 (-0.47 to 0.25)	-0.39 (-0.75 to 0.08)	0.27 (-0.02 to 0.63)	0.01 (-0.42 to 0.43)	0.31 (0.06 to 0.63)	0.53 (0.12 to 0.80)	-0.13 (-0.58 to 0.37)	0.48 (0.08 to 0.78)
DXA(% fat)	-0.09 (-0.48 to 0.32)	-0.32 (-0.61 to 0.06)	-0.26 (-0.61 to 0.28)	0.49 (0.15 to 0.74)	-0.02 (-0.29 to 0.23)	0.52 (0.20 to 0.74)	0.44 (-0.12 to 0.79)	0.20 (-0.21 to 0.48)	0.63 (-0.03 to 0.87)
DXA(bone)	-0.07 (-0.48 to 0.30)	-0.01 (-0.33 to 0.34)	-0.07 (-0.52 to 0.43)	-0.21 (-0.52 to 0.14)	-0.14 (-0.45 to 0.20)	-0.31 (-0.61 to 0.05)	-0.06 (-0.62 to 0.48)	-0.29 (-0.60 to 0.20)	-0.25 (-0.70 to 0.27)
MRI(subcutaneous)	-0.24 (-0.59 to 0.18)	-0.38 (-0.68 to 0.01)	-0.38 (-0.71 to 0.19)	0.55 (0.22 to 0.78)	-0.01 (-0.38 to 0.32)	0.64 (0.38 to 0.81)	0.48 (-0.13 to 0.80)	-0.01 (-0.47 to 0.47)	0.56 (-0.19 to 0.87)
MRI(abdominal)	-0.23 (-0.57 to 0.19)	-0.33 (-0.65 to 0.08)	-0.28 (-0.63 to 0.28)	0.49 (0.09 to 0.77)	0.01 (-0.39 to 0.37)	0.57 (0.27 to 0.77)	0.42 (-0.16 to 0.77)	0.10 (-0.37 to 0.54)	0.53 (-0.21 to 0.83)
MRI(total fat)	-0.17 (-0.53 to 0.22)	-0.21 (-0.61 to 0.20)	-0.08 (-0.50 to 0.42)	0.32 (-0.13 to 0.71)	0.04 (-0.44 to 0.41)	0.37 (0.04 to 0.67)	0.24 (-0.25 to 0.66)	0.21 (-0.25 to 0.61)	0.38 (-0.23 to 0.75)

$p < 0.05$; $p > 0.05$; $p = 0.05$. VAS: Visual assessment score; PDA: percent dense area; PDV: percent dense volume; FA: fat area; FV: fat volume; DA: dense area; DV: dense volume; TA: total area; TV: total volume; sqrt: square root transformed; cbrt: cube root transformed; BMI: body mass index; Imped: impedance; 95%CI: 95% confidence interval. Area-based measures from Cumulus; volumetric measures from Manchester Stepwedge. Within women effects represent trends over the entire 2 year period.

Table S3. Complete results for repeated measures between women correlations for different body composition measures.

Body Composition Measure	Weight (95%CI)	BMI (95%CI)	Waist (95%CI)	Imped (total fat) (95%CI)	Imped (% fat) (95%CI)	Imped (lean) (95%CI)	DXA (total fat) (95%CI)
Weight							
BMI	0.92 (0.88 to 0.95)						
Waist	0.87 (0.81 to 0.91)	0.87 (0.81 to 0.92)					
Imped(total fat)	0.98 (0.98 to 0.99)	0.94 (0.91 to 0.96)	0.89 (0.84 to 0.93)				
Imped(% fat)	0.91 (0.87 to 0.94)	0.89 (0.85 to 0.94)	0.87 (0.82 to 0.91)	0.96 (0.94 to 0.97)			
Imped(lean)	0.93 (0.88 to 0.96)	0.79 (0.65 to 0.88)	0.72 (0.61 to 0.82)	0.85 (0.77 to 0.91)	0.71 (0.58 to 0.81)		
DXA(total fat)	0.95 (0.93 to 0.97)	0.94 (0.91 to 0.97)	0.89 (0.83 to 0.93)	0.97 (0.96 to 0.98)	0.94 (0.92 to 0.97)	0.82 (0.70 to 0.90)	
DXA(lean)	0.88 (0.81 to 0.92)	0.72 (0.59 to 0.81)	0.64 (0.50 to 0.75)	0.82 (0.72 to 0.89)	0.70 (0.56 to 0.80)	0.93 (0.90 to 0.96)	0.70 (0.58 to 0.79)
DXA(% fat)	0.75 (0.65 to 0.83)	0.81 (0.75 to 0.87)	0.80 (0.71 to 0.88)	0.81 (0.73 to 0.88)	0.87 (0.81 to 0.91)	0.50 (0.30 to 0.67)	0.90 (0.86 to 0.94)
DXA(bone)	0.31 (0.11 to 0.50)	0.18 (-0.03 to 0.40)	0.11 (-0.11 to 0.34)	0.27 (0.07 to 0.47)	0.25 (0.02 to 0.46)	0.40 (0.18 to 0.61)	0.17 (-0.04 to 0.38)
MRI(subcutaneous)	0.86 (0.80 to 0.92)	0.90 (0.85 to 0.94)	0.89 (0.85 to 0.94)	0.88 (0.84 to 0.93)	0.87 (0.83 to 0.92)	0.72 (0.55 to 0.84)	0.93 (0.88 to 0.96)
MRI(abdominal)	0.87 (0.82 to 0.92)	0.92 (0.88 to 0.96)	0.93 (0.89 to 0.96)	0.90 (0.86 to 0.94)	0.89 (0.85 to 0.92)	0.73 (0.58 to 0.85)	0.93 (0.89 to 0.96)
MRI(total fat)	0.77 (0.69 to 0.88)	0.84 (0.77 to 0.90)	0.88 (0.80 to 0.93)	0.80 (0.72 to 0.90)	0.79 (0.71 to 0.87)	0.66 (0.51 to 0.84)	0.80 (0.71 to 0.90)

$p < 0.05$; $p > 0.05$; $p = 0.05$. BMI: body mass index; Imped: impedance; 95%CI: 95% confidence interval.

Table S3. (continued): Complete results for repeated measures between women correlations for different body composition measures.

Body Composition Measure	DXA (lean) (95%CI)	DXA (% fat) (95%CI)	DXA (bone) (95%CI)	MRI (subcutaneous) (95%CI)	MRI (abdominal) (95%CI)	MRI (total fat) (95%CI)
Weight						
BMI						
Waist						
Imped(total fat)						
Imped(% fat)						
Imped(lean)						
DXA(total fat)						
DXA(lean)						
DXA(% fat)	0.36 (0.18 to 0.53)					
DXA(bone)	0.47 (0.30 to 0.62)	-0.03 (-0.27 to 0.21)				

MRI(subcutaneous)	0.58 (0.44 to 0.70)	0.86 (0.79 to 0.90)	0.16 (-0.07 to 0.39)			
MRI(abdominal)	0.61 (0.48 to 0.71)	0.85 (0.78 to 0.90)	0.13 (-0.09 to 0.37)	0.98 (0.97 to 0.99)		
MRI(total fat)	0.57 (0.42 to 0.73)	0.71 (0.60 to 0.82)	0.06 (-0.15 to 0.32)	0.80 (0.72 to 0.89)	0.90 (0.86 to 0.94)	

$p < 0.05$; $p > 0.05$; $p = 0.05$. BMI: body mass index; Imped: impedance; 95%CI: 95% confidence interval.

Table S4. Complete results for repeated measures between women correlations for different mammographic density measures.

**Mammographic Density Measure**	**VAS (95%CI) (sqrt%)**	**PDA (95%CI) (sqrt%)**	**PDV (95%CI) (cbrt%)**	**FA (95%CI) (sqrt)**	**DA (95%CI) (sqrt)**	**TA (95%CI) (sqrt)**	**FV (95%CI) (cbrt)**	**DV (95%CI) (cbrt)**	**TV (95%CI) (cbrt)**
VAS (sqrt%)									
PDA (sqrt%)	0.90 (0.83 to 0.95)								
PDV (cbrt%)	0.79 (0.70 to 0.86)	0.78 (0.71 to 0.85)							
FA (sqrt)	-0.83 (-0.89 to -0.77)	-0.84 (-0.90 to -0.78)	-0.67 (-0.77 to -0.56)						
DA (sqrt)	0.50 (0.30 to 0.67)	0.68 (0.51 to 0.80)	0.48 (0.29 to 0.64)	-0.23 (-0.42 to -0.01)					
TA (sqrt)	-0.67 (-0.77 to -0.54)	-0.62 (-0.75 to -0.46)	-0.48 (-0.64 to -0.28)	0.94 (0.89 to 0.97)	0.11 (-0.10 to 0.34)				
FV (cbrt)	-0.81 (-0.88 to -0.73)	-0.74 (-0.83 to -0.63)	-0.72 (-0.81 to -0.60)	0.94 (0.92 to 0.97)	-0.10 (-0.30 to 0.12)	0.92 (0.87 to 0.95)			
DV (cbrt)	-0.09 (-0.31 to 0.14)	0.02 (-0.20 to 0.24)	0.30 (0.05 to 0.52)	0.42 (0.23 to 0.58)	0.55 (0.32 to 0.74)	0.66 (0.54 to 0.75)	0.43 (0.23 to 0.62)		
TV (cbrt)	-0.71 (-0.81 to -0.59)	-0.63 (-0.75 to -0.49)	-0.55 (-0.69 to -0.37)	0.92 (0.87 to 0.95)	0.05 (-0.16 to 0.28)	0.96 (0.93 to 0.98)	0.97 (0.95 to 0.98)	0.63 (0.49 to 0.75)	

$p < 0.05$; $p > 0.05$; $p = 0.05$. VAS: Visual assessment score; PDA: percent dense area; PDV: percent dense volume; FA: fat area; FV: fat volume; DA: dense area; DV: dense volume; TA: total area; TV: total volume; sqrt: square root transformed; cbrt: cube root transformed; 95%CI: 95% confidence interval. Area-based measures from Cumulus; volumetric measures from Manchester Stepwedge.

Table S5. Multivariable linear mixed model fit results for A1 using either body mass index or weight.

Mammographic Density Measure	BMI		Weight	
	AIC	BIC	AIC	BIC
VAS (sqrt%)	223.88	242.87	231.31	250.30
PDA (sqrt%)	307.27	326.19	315.67	334.59
PDV (cbrt%)	354.52	372.74	358.97	377.20
FA (sqrt)	167.56	186.61	174.90	193.95
FV (cbrt)	188.76	206.94	192.13	210.31
DA (sqrt)	373.07	392.13	373.80	392.86
DV (cbrt)	324.50	342.69	318.74	336.92

VAS: Visual assessment score; PDA: percent dense area; PDV: percent dense volume; FA: fat area; FV: fat volume; DA: dense area; DV: dense volume; sqrt: square root transformed; cbrt: cube root transformed; BMI: body mass index; AIC: Akaike information criterion; BIC: Bayesian information criterion. Area-based measures from Cumulus; volumetric measures from Manchester Stepwedge. Model: density on age at baseline and BMI (between and within) or weight (between and within), with a random per-woman intercept. Between women BMI calculated as the mean BMI for each woman; within women BMI calculated as the difference between each woman's BMI and her mean BMI. Between women weight calculated as the mean weight for each woman; within women weight calculated as the difference between each woman's weight and her mean weight.

Table S6. Repeated measures between women and within women correlations for mammographic density and body mass index, stratified by intervention group.

Intervention	Field	VAS (95%CI) (sqrt%)	PDA (95%CI) (sqrt%)	PDV (95%CI) (cbrt%)	FA (95%CI) (sqrt)	FV (95%CI) (cbrt)	DA (95%CI) (sqrt)	DV (95%CI) (cbrt)
Supervised weight loss programme	Cross-sectional BMI (between women)	-0.58 (-0.75 to -0.36)	-0.51 (-0.72 to -0.26)	-0.50 (-0.72 to -0.28)	0.74 (0.58 to 0.86)	0.78 (0.69 to 0.89)	0.16 (-0.17 to 0.48)	0.56 (0.28 to 0.76)
	Short-term BMI change (within women)	-0.20 (-0.53 to 0.15)	-0.30 (-0.58 to 0.04)	-0.42 (-0.65 to -0.10)	0.53 (0.21 to 0.75)	0.70 (0.41 to 0.84)	-0.05 (-0.38 to 0.28)	0.07 (-0.25 to 0.35)
Usual care	Cross-sectional BMI (between women)	-0.69 (-0.83 to -0.47)	-0.69 (-0.85 to -0.43)	-0.49 (-0.67 to -0.27)	0.74 (0.56 to 0.86)	0.75 (0.59 to 0.86)	-0.42 (-0.71 to 0.00)	0.22 (-0.15 to 0.56)
	Short-term BMI change (within women)	-0.35 (-0.61 to -0.09)	-0.14 (-0.46 to 0.19)	-0.27 (-0.55 to 0.20)	0.35 (0.05 to 0.62)	0.46 (0.03 to 0.74)	0.07 (-0.30 to 0.43)	0.10 (-0.32 to 0.41)

VAS: Visual assessment score; PDA: percent dense area; PDV: percent dense volume; FA: fat area; FV: fat volume; DA: dense area; DV: dense volume; sqrt: square root transformed; cbrt: cube root transformed; BMI: body mass index; 95%CI: 95% confidence interval. Area-based measures from Cumulus; volumetric measures from Manchester Stepwedge. Within women correlations represent trends over the entire 2 year period.

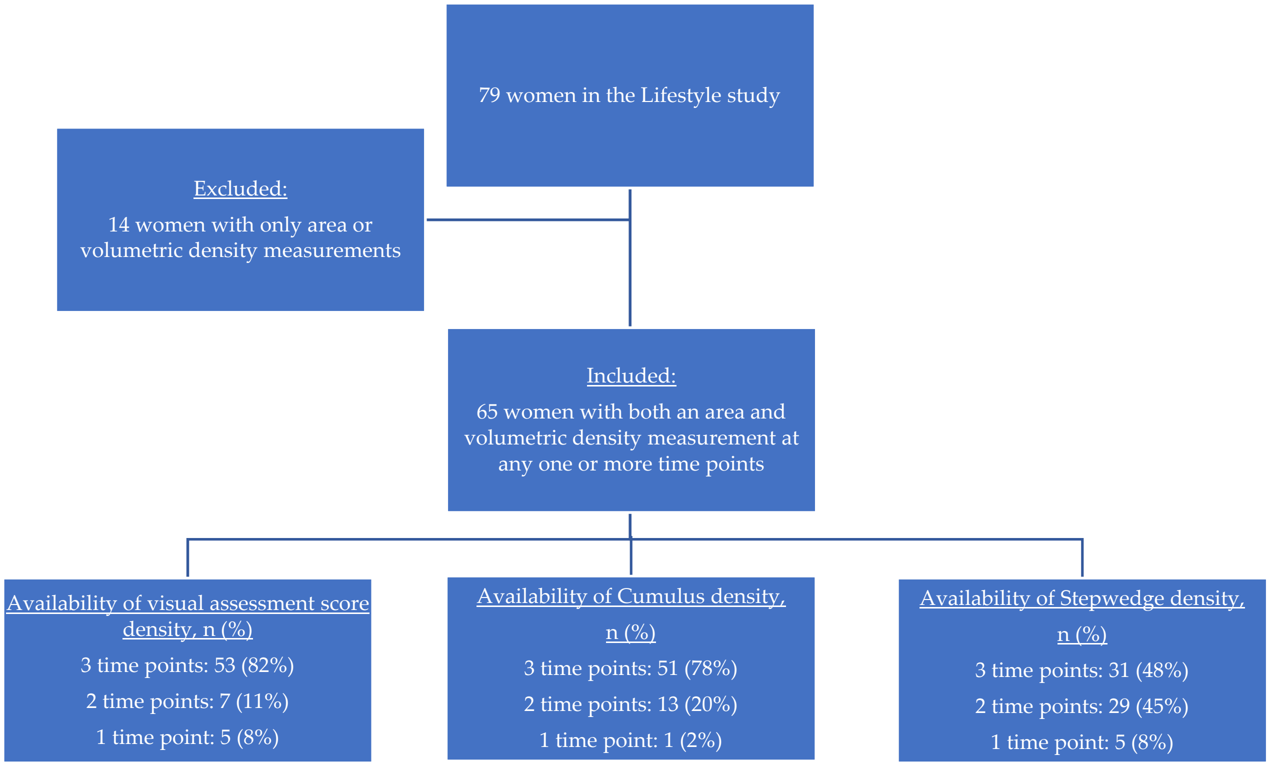

Figure S1. Flow chart of women included in the analysis and availability of mammographic density data. All women had weight and body mass index (BMI) available at all time-points except for one woman with missing weight and BMI at 2 years (data point excluded from analyses involving weight or BMI).
